# A quantitative analysis of secondary RNA structure using domination based parameters on trees

**DOI:** 10.1186/1471-2105-7-108

**Published:** 2006-03-03

**Authors:** Teresa Haynes, Debra Knisley, Edith Seier, Yue Zou

**Affiliations:** 1 Mathematics and Statistics Department, Box 70663, East Tennessee State University, Johnson City, TN, USA; 2 Department of Biochemistry and Molecular Biology, Quillen College of Medicine, East Tennessee State University, Johnson City, TN, USA

## Abstract

**Background:**

It has become increasingly apparent that a comprehensive database of RNA motifs is essential in order to achieve new goals in genomic and proteomic research. Secondary RNA structures have frequently been represented by various modeling methods as graph-theoretic trees. Using graph theory as a modeling tool allows the vast resources of graphical invariants to be utilized to numerically identify secondary RNA motifs. The domination number of a graph is a graphical invariant that is sensitive to even a slight change in the structure of a tree. The invariants selected in this study are variations of the domination number of a graph. These graphical invariants are partitioned into two classes, and we define two parameters based on each of these classes. These parameters are calculated for all small order trees and a statistical analysis of the resulting data is conducted to determine if the values of these parameters can be utilized to identify which trees of orders seven and eight are RNA-like in structure.

**Results:**

The statistical analysis shows that the domination based parameters correctly distinguish between the trees that represent native structures and those that are not likely candidates to represent RNA. Some of the trees previously identified as candidate structures are found to be "very" RNA like, while others are not, thereby refining the space of structures likely to be found as representing secondary RNA structure.

**Conclusion:**

Search algorithms are available that mine nucleotide sequence databases. However, the number of motifs identified can be quite large, making a further search for similar motif computationally difficult. Much of the work in the bioinformatics arena is toward the development of better algorithms to address the computational problem. This work, on the other hand, uses mathematical descriptors to more clearly characterize the RNA motifs and thereby reduce the corresponding search space. These preliminary findings demonstrate that graph-theoretic quantifiers utilized in fields such as computer network design hold significant promise as an added tool for genomics and proteomics.

## Background

Predicting the final fold of RNA from its sequence is a challenging problem, but has played a secondary role to the protein structure prediction problem. Interest in both the prediction of secondary and tertiary RNA structure is currently gaining substantial momentum. Recently, the Journal *Science *devoted a special issue to the form and function of RNA [1]. It is now known that RNA is involved in a large variety of processes, including gene regulation. Despite this, the important task of classifying RNA molecules in order to identify structural motifs remains far from complete. Many classes of RNA molecules are characterized by highly conserved secondary structures. Since RNA molecules maintain independently stable and highly conserved secondary folds, RNA function is also highly correlated with its secondary structure. Thus, we focus on identifying structural characteristics of secondary RNA.

The utility of graphs as models of proteins and nucleic acids is fertile ground for the discovery of new and innovative methods for the numerical characterization of biomolecules. In this paper we address the applicability of graphs in the analysis of secondary RNA structure. A mathematical graph, or simply a graph, is a set of points, called vertices, and connecting lines, called edges. Trees are a familiar example of graphs since they are used extensively to aid in phylogenetic studies. RNA tree graphs were first developed by Le et al.[2] and Benededetti and Morosetti[3] to determine structural similarities in RNA. Secondary structure tree representation can also be found in Waterman's classic text, *An Introduction to Computational Biology*[4]. In a recent paper titled *Exploring the repertoire of RNA secondary motifs using graph theory; implications for RNA design*, researchers led by Tamar Schlick developed a new method for representing secondary RNA structure as a two dimensional RNA tree graph[5]. Unlike the classic model developed by Waterman et.al. where atoms are represented by vertices and bonds between the atoms by edges in the graph, the RAG (RNA as Graphs) project represents stems as edges and breaks in the stems that result in bulges and loops as vertices. A nucleotide bulge, hairpin loop or internal loop are each represented by a vertex when there is more than one unmatched nucleotide or non-complementary base pair. This modeling method is illustrated in figures 8 and 9. Their method has led to the creation of an RNA topology database called RAG (Rna As Graphs) that is published and available at BMC Bioinformatics and Bioinformatics[6,7]. In this database, all possible unlabeled trees of a given order (number of vertices) are presented for orders two through eleven. For trees of order eight and below, a color scheme is used; red trees represent a known native secondary RNA structure, blue trees are listed as likely candidates and black trees are those structures that are considered not likely to be found as RNA structures. For trees of order nine and above, blue is not utilized. That is, the likely candidates are not identified. In this work we demonstrate that a graphical analysis of the trees that have been classified by the color scheme, without the aid of thermodynamic properties of the nucleic acids or other biophysical considerations, can determine which trees are RNA-like in structure.

The total number of possible RNA tree graphs for a given number of vertices is given by the tree enumeration theorems of Harary and Prins[8]. Schlick et al.[5] found that existing RNA classes represent only a small subset of the possible tree representations of two-dimensional RNA motifs. It is believed that many more will either be found as a native structure, or synthetically developed. Thus, investigating the quantitative properties of the trees not known to exist as native structures is a natural way to proceed. In a successive paper by the Schlick group, candidates for novel RNA topologies where identified [9]. The RAG project uses two representations for secondary RNA; trees as described above, and dual graphs which we have not discussed here. In [8], dual graphs are used in the analysis and in this work, we analyze the tree graphs. Dual graphs have the advantage in that all secondary RNA structures have a dual graph representation, whereas only certain RNA structures can be represented as a tree. However, part of the purpose of this work is to test the applicability of the enormous amount of graphical invariants available that might aid in the quantification of biomolecules. This work demonstrates the potential for this line of investigation and in fact shows that invariants used in network design and fault-tolerant computing lend themselves to a quantitative analysis of secondary RNA structures.

## Results

### Graph-theoretic analysis

**Figure 1:**
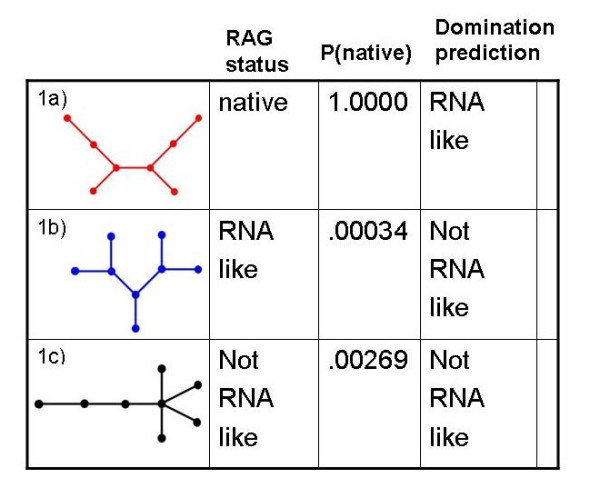
**Three trees of order 8 **has three trees of order 8, Figure 1a, 1b and figure 1c.

In the RNA database RAG, the trees are catalogued by their Fiedler (second smallest) eigenvalue, denoted by *λ*_2_[6]. The trees are listed in increasing order, the tree with the smallest value of *λ*_2 _first and the tree with the largest last. The trees are labeled by the number of vertices in the tree followed by their order of listing. The tree with eight vertices in figure 1a is labeled 8.11 in the RAG database and models a native structure. The tree in 1b is labeled 8.13 and is indicated by RAG as a candidate (RNA-like) structure, and the tree in figure 1c is labeled 8.14 and is classified as not RNA-like in structure by RAG. The candidate tree structure in figure 1b is not predicted to be RNA-like by the domination parameter models. However the domination based models agree with the database on all of the other trees of order eight. In this paper we determine five domination numbers for each tree, namely the domination, total domination, global alliance, locating-dominating and differentiation domination numbers. These graphical invariants are defined in the section on graph theory definitions and terminology. The domination number of each of the three trees in figure 1 are four, three and two respectively, placing the candidate numerically between the known native structure and the structure classified in the RAG database as not RNA-like. With respect to the total domination number and the global alliance number, there is little or no indication of any variation in the classification. That is, the domination numbers for the tree in figure 1b are indicative that the tree's efficiency (with respect to the domination numbers) is between the RNA-like and not RNA-like trees. However, with respect to the locating-dominating number, the tree in figure 1b behaves very much like the tree in figure 1c. In particular, the candidate's structure, when viewed in terms of this particular domination invariant, is highly inefficient as is the not RNA-like tree in 1c. In some sense, one could say that the domination based parameters reveal an underlying efficient communication network. Clearly, a single graphical invariant such as the Fiedler eigenvalue or the locating-dominating number is not sufficient to numerically characterize biological molecules. However, by defining parameters that combine these measures, we develop a meaningful prediction tool of the native structures. Accordingly, we define two parameters *p*_1 _and *P*_2 _by the graphical invariants we define later in this paper. We also modify *P*_2 _by the Fiedler eigenvalues to further investigate this parameter and denote it by P2*
 MathType@MTEF@5@5@+=feaafiart1ev1aaatCvAUfKttLearuWrP9MDH5MBPbIqV92AaeXatLxBI9gBaebbnrfifHhDYfgasaacH8akY=wiFfYdH8Gipec8Eeeu0xXdbba9frFj0=OqFfea0dXdd9vqai=hGuQ8kuc9pgc9s8qqaq=dirpe0xb9q8qiLsFr0=vr0=vr0dc8meaabaqaciaacaGaaeqabaqabeGadaaakeaacqWGqbaudaqhaaWcbaGaeGOmaidabaGaeiOkaOcaaaaa@2FD0@.

### Statistical results

The results from the statistical analysis are shown in Table 1. The objective of the statistical analysis was to explore the possibility of predicting if a tree is RNA-like based on the values of the two variables defined solely in terms of domination *P*_1 _= (*γ *+ *γ*_*t *_+ *γ*_*a*_)/*n *(domination + total domination + global alliance)/n and *P*_2 _= (*γ*_*L *_+ *γ*_*D*_)/*n*(locating domination + differentiating domination)/n. As an alternative for *P*_2_, a third variable was considered P2*
 MathType@MTEF@5@5@+=feaafiart1ev1aaatCvAUfKttLearuWrP9MDH5MBPbIqV92AaeXatLxBI9gBaebbnrfifHhDYfgasaacH8akY=wiFfYdH8Gipec8Eeeu0xXdbba9frFj0=OqFfea0dXdd9vqai=hGuQ8kuc9pgc9s8qqaq=dirpe0xb9q8qiLsFr0=vr0=vr0dc8meaabaqaciaacaGaaeqabaqabeGadaaakeaacqWGqbaudaqhaaWcbaGaeGOmaidabaGaeiOkaOcaaaaa@2FD0@ that also incorporates the second smallest eigenvalue of the graph, P2*
 MathType@MTEF@5@5@+=feaafiart1ev1aaatCvAUfKttLearuWrP9MDH5MBPbIqV92AaeXatLxBI9gBaebbnrfifHhDYfgasaacH8akY=wiFfYdH8Gipec8Eeeu0xXdbba9frFj0=OqFfea0dXdd9vqai=hGuQ8kuc9pgc9s8qqaq=dirpe0xb9q8qiLsFr0=vr0=vr0dc8meaabaqaciaacaGaaeqabaqabeGadaaakeaacqWGqbaudaqhaaWcbaGaeGOmaidabaGaeiOkaOcaaaaa@2FD0@ = *γ*_*L *_+ *γ*_*D *_+ *n λ*_2 _(locating domination + differentiating domination + n (second smallest eigenvalue)). Separate dotplots were prepared for 7 and 8 vertices trees, both for *P*_1_, *P*_2 _and P2*
 MathType@MTEF@5@5@+=feaafiart1ev1aaatCvAUfKttLearuWrP9MDH5MBPbIqV92AaeXatLxBI9gBaebbnrfifHhDYfgasaacH8akY=wiFfYdH8Gipec8Eeeu0xXdbba9frFj0=OqFfea0dXdd9vqai=hGuQ8kuc9pgc9s8qqaq=dirpe0xb9q8qiLsFr0=vr0=vr0dc8meaabaqaciaacaGaaeqabaqabeGadaaakeaacqWGqbaudaqhaaWcbaGaeGOmaidabaGaeiOkaOcaaaaa@2FD0@. However since they exhibit a similar behavior, they were combined into a single dotplot for both sets of trees. In the individual dotplots it was observed that the gap in *P*_1 _value between the native trees and the trees not likely to represent RNA was wider for the 7 vertices trees than for the 8 vertices trees; but in both cases, all native trees had *P*_1 _> 1.2. In a similar fashion, the gap between the *P*_2 _and P2*
 MathType@MTEF@5@5@+=feaafiart1ev1aaatCvAUfKttLearuWrP9MDH5MBPbIqV92AaeXatLxBI9gBaebbnrfifHhDYfgasaacH8akY=wiFfYdH8Gipec8Eeeu0xXdbba9frFj0=OqFfea0dXdd9vqai=hGuQ8kuc9pgc9s8qqaq=dirpe0xb9q8qiLsFr0=vr0=vr0dc8meaabaqaciaacaGaaeqabaqabeGadaaakeaacqWGqbaudaqhaaWcbaGaeGOmaidabaGaeiOkaOcaaaaa@2FD0@ values between native structures and those not likely to represent RNA was wider in the 7 vertices trees; but in both cases, the native trees have lower values of *P*_2_.

**Figure 2:**
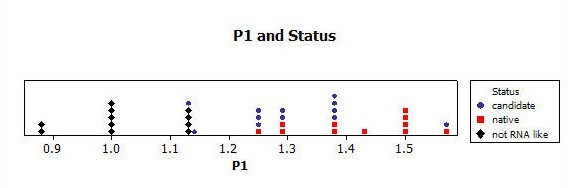
Dot plot for *P*_1_.

**Figure 3:**
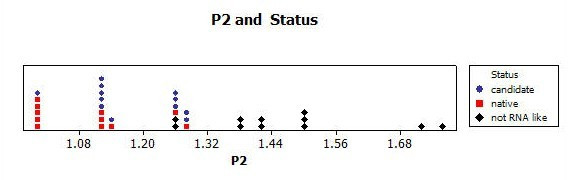
Dot plot for *P*_2_.

**Figure 4:**
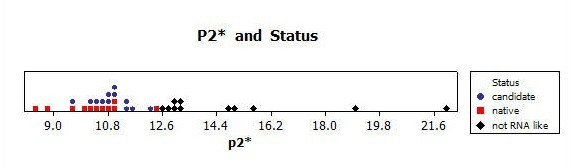
Dot plot for P2*
 MathType@MTEF@5@5@+=feaafiart1ev1aaatCvAUfKttLearuWrP9MDH5MBPbIqV92AaeXatLxBI9gBaebbnrfifHhDYfgasaacH8akY=wiFfYdH8Gipec8Eeeu0xXdbba9frFj0=OqFfea0dXdd9vqai=hGuQ8kuc9pgc9s8qqaq=dirpe0xb9q8qiLsFr0=vr0=vr0dc8meaabaqaciaacaGaaeqabaqabeGadaaakeaacqWGqbaudaqhaaWcbaGaeGOmaidabaGaeiOkaOcaaaaa@2FD0@.

**Figure 5:**
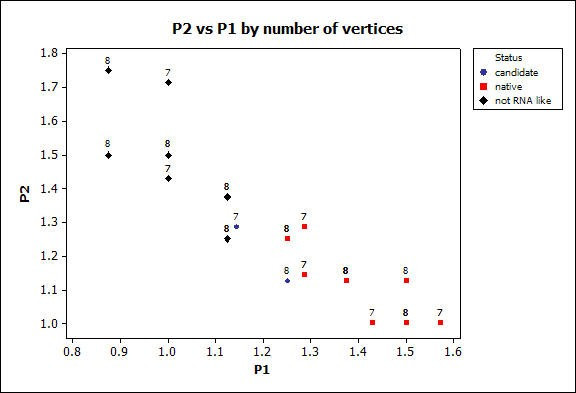
Scatter Plot for *P*_1 _vs *P*_2_.

**Figure 6:**
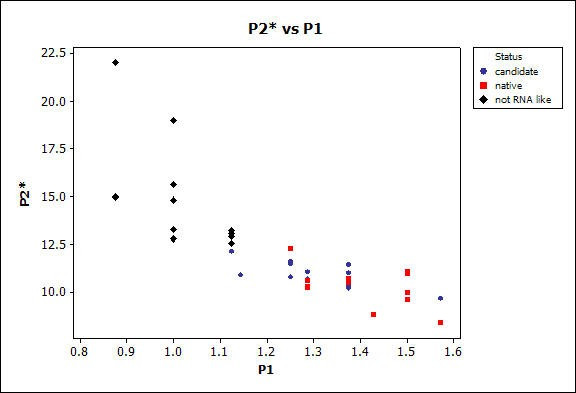
Scatter Plot for *P*_1 _vs P2*
 MathType@MTEF@5@5@+=feaafiart1ev1aaatCvAUfKttLearuWrP9MDH5MBPbIqV92AaeXatLxBI9gBaebbnrfifHhDYfgasaacH8akY=wiFfYdH8Gipec8Eeeu0xXdbba9frFj0=OqFfea0dXdd9vqai=hGuQ8kuc9pgc9s8qqaq=dirpe0xb9q8qiLsFr0=vr0=vr0dc8meaabaqaciaacaGaaeqabaqabeGadaaakeaacqWGqbaudaqhaaWcbaGaeGOmaidabaGaeiOkaOcaaaaa@2FD0@.

**Figure 7:**
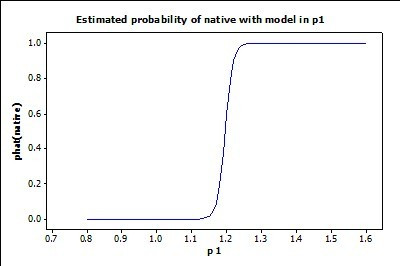
Graph of estimated probabilities for *P*_1_.

**Figure 8:**
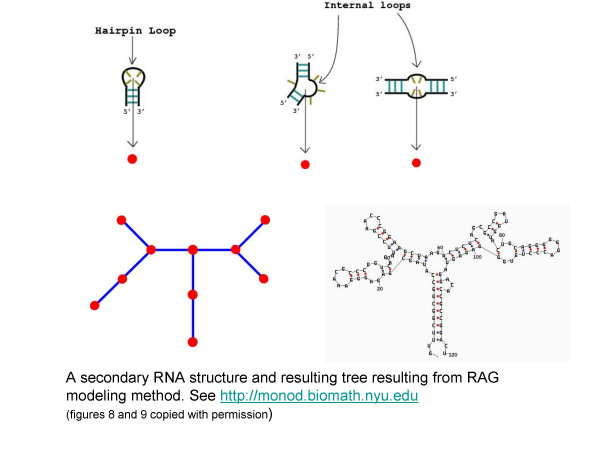


**Figure 9:**
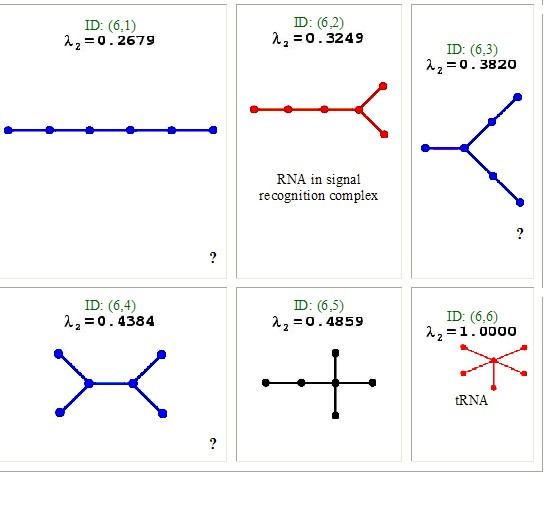


The dot-plots in Figures 2, 3, and 4 for *P*_1_, *P*_2 _and P2*
 MathType@MTEF@5@5@+=feaafiart1ev1aaatCvAUfKttLearuWrP9MDH5MBPbIqV92AaeXatLxBI9gBaebbnrfifHhDYfgasaacH8akY=wiFfYdH8Gipec8Eeeu0xXdbba9frFj0=OqFfea0dXdd9vqai=hGuQ8kuc9pgc9s8qqaq=dirpe0xb9q8qiLsFr0=vr0=vr0dc8meaabaqaciaacaGaaeqabaqabeGadaaakeaacqWGqbaudaqhaaWcbaGaeGOmaidabaGaeiOkaOcaaaaa@2FD0@ respectively show that native structures tend to have high values of *P*_1 _and low values of either *P*_2 _or P2*
 MathType@MTEF@5@5@+=feaafiart1ev1aaatCvAUfKttLearuWrP9MDH5MBPbIqV92AaeXatLxBI9gBaebbnrfifHhDYfgasaacH8akY=wiFfYdH8Gipec8Eeeu0xXdbba9frFj0=OqFfea0dXdd9vqai=hGuQ8kuc9pgc9s8qqaq=dirpe0xb9q8qiLsFr0=vr0=vr0dc8meaabaqaciaacaGaaeqabaqabeGadaaakeaacqWGqbaudaqhaaWcbaGaeGOmaidabaGaeiOkaOcaaaaa@2FD0@. The scatter-plots in Figures 5 and 6 also show that there is a strong negative correlation between *P*_1 _and either *P*_2 _or P2*
 MathType@MTEF@5@5@+=feaafiart1ev1aaatCvAUfKttLearuWrP9MDH5MBPbIqV92AaeXatLxBI9gBaebbnrfifHhDYfgasaacH8akY=wiFfYdH8Gipec8Eeeu0xXdbba9frFj0=OqFfea0dXdd9vqai=hGuQ8kuc9pgc9s8qqaq=dirpe0xb9q8qiLsFr0=vr0=vr0dc8meaabaqaciaacaGaaeqabaqabeGadaaakeaacqWGqbaudaqhaaWcbaGaeGOmaidabaGaeiOkaOcaaaaa@2FD0@; r(P1,P2)
 MathType@MTEF@5@5@+=feaafiart1ev1aaatCvAUfKttLearuWrP9MDH5MBPbIqV92AaeXatLxBI9gBaebbnrfifHhDYfgasaacH8akY=wiFfYdH8Gipec8Eeeu0xXdbba9frFj0=OqFfea0dXdd9vqai=hGuQ8kuc9pgc9s8qqaq=dirpe0xb9q8qiLsFr0=vr0=vr0dc8meaabaqaciaacaGaaeqabaqabeGadaaakeaacqWGYbGCdaWgaaWcbaGaeiikaGIaemiuaa1aaSbaaWqaaiabigdaXaqabaWccqGGSaalcqWGqbaudaWgaaadbaGaeGOmaidabeaaliabcMcaPaqabaaaaa@357B@ = -0.92 and r(P1,P2*)
 MathType@MTEF@5@5@+=feaafiart1ev1aaatCvAUfKttLearuWrP9MDH5MBPbIqV92AaeXatLxBI9gBaebbnrfifHhDYfgasaacH8akY=wiFfYdH8Gipec8Eeeu0xXdbba9frFj0=OqFfea0dXdd9vqai=hGuQ8kuc9pgc9s8qqaq=dirpe0xb9q8qiLsFr0=vr0=vr0dc8meaabaqaciaacaGaaeqabaqabeGadaaakeaacqWGYbGCdaWgaaWcbaGaeiikaGIaemiuaa1aaSbaaWqaaiabigdaXaqabaWccqGGSaalcqWGqbaudaWgaaadbaGaeGOmaiJaeiOkaOcabeaaliabcMcaPaqabaaaaa@3657@ = -0.809. The correlation is slightly stronger for trees with eight vertices. The estimated probabilities *P*(*native*) are plotted for a range of values of *P*_1 _in Figure 7. The class (native or not RNA-like) predicted for the candidates is the same for both logistic models. The logistic models, described in the methods section of the paper, correctly identify all native structures and agree with the RAG database prediction with respect to the non RNA-like structures. However, it identifies two structures that are indicated as RNA-like in the RAG database as not RNA-like. The two RAG candidates that our model rejects can be easily spotted in Figure 2. Figure 2 also shows that one of the candidates, listed in the RAG database as a candidate is an "exceptionally good" candidate.

## Discussion

### RNA motifs

The RAG database classifies all possible tree structures with eight or fewer vertices as either native structures that have been found, candidate RNAs or non RNA-like in structure. Those that are RNA-like in structure that have not been verified as existing are considered candidates that may later be identified or artificially produced. In this study, we consider all of the tree structures with seven or eight vertices. Using the graphical parameters *P*_1_, *P*_2 _and P2*
 MathType@MTEF@5@5@+=feaafiart1ev1aaatCvAUfKttLearuWrP9MDH5MBPbIqV92AaeXatLxBI9gBaebbnrfifHhDYfgasaacH8akY=wiFfYdH8Gipec8Eeeu0xXdbba9frFj0=OqFfea0dXdd9vqai=hGuQ8kuc9pgc9s8qqaq=dirpe0xb9q8qiLsFr0=vr0=vr0dc8meaabaqaciaacaGaaeqabaqabeGadaaakeaacqWGqbaudaqhaaWcbaGaeGOmaidabaGaeiOkaOcaaaaa@2FD0@, our findings are consistent with the database. That is, the domination based parameters used in the logistic models identify two clusters. All of the native structures and almost all of the RAG candidate structures are predicted as RNA-like by our model and the structures identified by RAG as not RNA-like are also not RNA-like by our models. We also conclude that the tree labeled 8.16 is an exceptionally good candidate while the model rejects trees 7.4 and 8.13 (figure 1b). The emerging area of RNA as a tool and target has produced a wealth of new and innovative pharmaceutical applications. Chemically synthesized RNA's have been produced to aid in the development of novel therapeutics. Functional clusters of RNA, both mRNA and regulatory RNA binding proteins are a rich source of therapeutic tools for the management and potential cures of human disease. This novel approach for identifying tree structures that have definite RNA-like characteristics shows promise as an added tool for the design and analysis of nucleic acids.

### Graphs as mathematical objects

A graph is a mathematical object that is frequently described as a set of points (vertices) and a set of lines (edges) that connect some, possibly all, of the points. If two points in the graph are connected by a line, they are said to be adjacent, otherwise they are nonadjacent. How the lines are drawn, straight, curved, long, or short is irrelevant; only the connection is relevant. An alternate definition of a graph is a set of elements with a well-defined relation. Each element in the set can be represented by a point and if two elements in the set are related, then the corresponding points are connected by an edge. So the common definition of a graph is really a visual representation of a relationship that is defined on a set of elements. In graph theory, one then studies the relational representation as an object in its own right, discerning properties of the object and quantifying the results. These quantities are called graphical invariants since their values are the same regardless of how the graph is drawn. The graphical invariants, in turn, tell us about the consequences the relation has on the set. The domination number of a graph is an example of such an invariant. The idea of domination is based on sets of vertices that "are near" (dominate) all the vertices of a graph. To illustrate the definition of the domination number of a graph, we consider an example of its application. Suppose each vertex of the graph represents a computer and two computers are adjacent if there is a direct link between them in the network. Some of the computers are designated as file servers to house the programs for the entire network. If the file servers are selected in such a way that every computer is either a file server or has a direct connection to a file server, then the set of file servers is a dominating set. The minimum number of file servers required so that every computer in the network has access to one is the domination number of the associated graph. For more information on the domination number of graphs see[10]. There are numerous graphical invariants defined for graphs. Our selection of the invariants for the trees is based on those that are sensitive to a change in the structure of a tree. For example, the locating-domination number of a graph is defined as the minimum number of vertices in any locating-dominating set. A locating-dominating set of vertices with the following properties:

1. any vertex outside the set must be adjacent to at least one in the locating-dominating set.

2. given a a single vertex outside the dominating set, the set of vertices in the locating-dominating set that this single vertex is adjacent to is always unique.

If we think of two vertices in the tree as regions in the RNA structure where interaction is most likely to occur due to the fact that there are unpaired nucleotide bases, then if an "interaction" occurs, a mechanism is in place that makes it is possible to discern the location of the interacting region. Graphs have been used extensively to aid in the design and analysis of algorithms and hence are an integral part of the field of bioinformatics [11-13]. However, the use of graphs as the biomolecules themselves has been fairly limited. There have been some earlier models of biomolecules as graphs, but in those cases the graph's spectrum is the primary focus of the analysis[14-16]. For a nice survey on some of the applications see graphs and proteins see[17]. Spectral graph theory has been a useful tool for chemist who have used graphs to model molecules. And other graph theoretic measures have been defined that are well suited for molecular description in the spirit of chemical graph theory[18]. However, the field of graph theory offers many other tools and techniques for further quantification and analysis of graphs. In this work, we show that graphical invariants, which aid in the optimization of computer and electrical networks, are a remarkable new source of information about the structure of secondary RNA molecules.

## Conclusion

We have demonstrated that graphical invariants based on domination numbers can numerically identify characteristics of secondary RNA structure. Search algorithms such as RNAMotif[19] can be used to mine nucleotide sequence databases for motifs. RNAMotif allows users to identify similar motifs within the database. However, when the constraints are relaxed to provide more flexibility, the number of motifs identified by the algorithm may become very large. Exhaustive methods to search for similar RNA structure over these large search spaces are likely to be computationally intractable. Much of the work in the bioinformatics arena is toward the development of better algorithms to address the computational problem. This work, on the other hand, uses mathematical descriptors that can easily and more clearly characterize the RNA motifs and thereby significantly reduce the corresponding search space. The graphical invariants used to identify structural characteristics of a class of biomolecules depends on the corresponding graph. By representing biomolecules as graphs, we can then thoroughly investigate the graph using the appropriate graphical invariants; thereby quantifying the structure. Although determining graphical invariants in general is computationally difficult as well, for special classes of graphs such as trees there exist fast algorithms for their computation. These preliminary findings from this novel approach are intriguing and the method shows promise as an added tool for genomic and proteomic prediction tools.

## Methods

### Graph theory definitions and terminology

Trees have been highly studied as a family of graphs. Therefore, in this work, we employ graphical invariants that are indicative of variations in the structure of trees. In particular, we utilize a number of domination parameters that are highly sensitive to the structural changes of small ordered trees. First we define the graphical invariants that are utilized in this work. These definitions can be found in *Fundamentals of Domination in Graphs, Chemical Graph Theory *or in *Graph Theory and its Applications *[10,20,21] We denote the vertex set of a graph by *V*(*G*), or simply *V*. The number of edges incident to a vertex *v *is the *degree *of the vertex *deg*(*v*) and two vertices are *adjacent *if they are incident to the same edge. A vertex set *S *is a *dominating set *if for every vertex *u *∈ *V *- *S*, *u *is adjacent to at least one vertex in *S*. The *domination number γ*(*G*) is the minimum cardinality among all dominating sets in *G*. A set *S *is a *total dominating set *if for every vertex *u *∈ *V*, *u *is adjacent to at least one vertex in *S *(note here that even the vertices in *S *must be adjacent to a vertex in *S*). The *total domination number γ*_*t*_(*G*) is the minimum cardinality among all total dominating sets in *G*. The *neighborhood of a vertex v*, denoted by *N*(*v*), is the set of all vertices adjacent to *v *and the *closed neighborhood of a vertex u *is *N*[*u*] = *N*(*u*) ∪ {*u*}. A dominating set *S *is called a *locating-dominating set *if for any two vertices *v*, *w *∈ *V *- *S*, *N*(*v*) ∩ *S *≠ *N*(*w*) ∩ *S*. Thus, in a locating dominating set, every vertex in *V *- *S *is dominated by a distinct subset of the vertices of *S*. The *locating-domination number *of a graph *G *is the minimum cardinality among all locating dominating sets in *G *and is denoted by *γ*_*L*_(*G*). A dominating set *S *is called a *differentiating dominating set *if for any two vertices *v*, *w *∈ *V*, *N*[*v*] ∩ *S *≠ *N*[*w*] ∩ *S*. The *differentiating domination number *of a graph *G *is the minimum cardinality among all differentiating dominating sets in *G *and is denoted by *γ*_*D*_(*G*). The *global alliance number *of a graph *G *is the minimum cardinality among all global alliances of *G*, where a set *S *is a global alliance if *S *is a dominating set and for each *u *∈ *S*, the number of "allies" it has in *S *are at least as many as it has in *V *- *S*. In other words, *S *is a dominating set and for each vertex *u *∈ *S*, it is true that |*N*[*u*] ∩ *S*| ≥ |*N*(*u*) ∩ (*V *- *S*)|. The *adjacency matrix A *= *A*(*G*) and the *degree matrix D *= *D*(*G*) are the square matrices that contain information about the internal connectivity of vertices in *G*. They are defined as

Ai,j={1if and only if vi and vj are adjacent0otherwise
 MathType@MTEF@5@5@+=feaafiart1ev1aaatCvAUfKttLearuWrP9MDH5MBPbIqV92AaeXatLxBI9gBaebbnrfifHhDYfgasaacH8akY=wiFfYdH8Gipec8Eeeu0xXdbba9frFj0=OqFfea0dXdd9vqai=hGuQ8kuc9pgc9s8qqaq=dirpe0xb9q8qiLsFr0=vr0=vr0dc8meaabaqaciaacaGaaeqabaqabeGadaaakeaacqWGbbqqdaWgaaWcbaGaemyAaKMaeiilaWIaemOAaOgabeaakiabg2da9maaceaabaqbaeaabiGaaaqaaiabigdaXaqaaiabbMgaPjabbAgaMjabbccaGiabbggaHjabb6gaUjabbsgaKjabbccaGiabb+gaVjabb6gaUjabbYgaSjabbMha5jabbccaGiabbMgaPjabbAgaMjabbccaGiabbAha2naaBaaaleaacqWGPbqAaeqaaOGaeeiiaaIaeeyyaeMaeeOBa4MaeeizaqMaeeiiaaIaemODay3aaSbaaSqaaiabdQgaQbqabaGccqqGGaaicqqGHbqycqqGYbGCcqqGLbqzcqqGGaaicqqGHbqycqqGKbazcqqGQbGAcqqGHbqycqqGJbWycqqGLbqzcqqGUbGBcqqG0baDaeaacqaIWaamaeaacqqGVbWBcqqG0baDcqqGObaAcqqGLbqzcqqGYbGCcqqG3bWDcqqGPbqAcqqGZbWCcqqGLbqzaaaacaGL7baaaaa@6FE8@

Di,j={deg(vi)ifi=j0otherwise
 MathType@MTEF@5@5@+=feaafiart1ev1aaatCvAUfKttLearuWrP9MDH5MBPbIqV92AaeXatLxBI9gBaebbnrfifHhDYfgasaacH8akY=wiFfYdH8Gipec8Eeeu0xXdbba9frFj0=OqFfea0dXdd9vqai=hGuQ8kuc9pgc9s8qqaq=dirpe0xb9q8qiLsFr0=vr0=vr0dc8meaabaqaciaacaGaaeqabaqabeGadaaakeaacqWGebardaWgaaWcbaGaemyAaKMaeiilaWIaemOAaOgabeaakiabg2da9maaceaabaqbaeaabiWaaaqaaGqaciab=rgaKjab=vgaLjab=DgaNjabcIcaOiabdAha2naaBaaaleaacqWGPbqAaeqaaOGaeiykaKcabaGaeeyAaKMaeeOzaygabaGaemyAaKMaeyypa0JaemOAaOgabaGaeGimaadabaGaee4Ba8MaeeiDaqNaeeiAaGMaeeyzauMaeeOCaiNaee4DaCNaeeyAaKMaee4CamNaeeyzaugabaaaaaGaay5Eaaaaaa@503A@

The *Laplacian matrix L *= *L*_*ij*_(*G*) is the square matrix defined by *L *= *D *- *A*

Lij={deg(vi)ifi=j−1ifi≠j and (vi,vj)∈E(G)0otherwise
 MathType@MTEF@5@5@+=feaafiart1ev1aaatCvAUfKttLearuWrP9MDH5MBPbIqV92AaeXatLxBI9gBaebbnrfifHhDYfgasaacH8akY=wiFfYdH8Gipec8Eeeu0xXdbba9frFj0=OqFfea0dXdd9vqai=hGuQ8kuc9pgc9s8qqaq=dirpe0xb9q8qiLsFr0=vr0=vr0dc8meaabaqaciaacaGaaeqabaqabeGadaaakeaacqWGmbatdaWgaaWcbaGaemyAaKMaemOAaOgabeaakiabg2da9maaceaabaqbaeaabmWaaaqaaGqaciab=rgaKjab=vgaLjab=DgaNjabcIcaOiabdAha2naaBaaaleaacqWGPbqAaeqaaOGaeiykaKcabaGaeeyAaKMaeeOzaygabaGaemyAaKMaeyypa0JaemOAaOgabaGaeyOeI0IaeGymaedabaGaeeyAaKMaeeOzaygabaGaemyAaKMaeyiyIKRaemOAaOMaeeiiaaIaeeyyaeMaeeOBa4MaeeizaqMaeeiiaaIaeiikaGIaemODay3aaSbaaSqaaiabdMgaPbqabaGccqGGSaalcqWG2bGDdaWgaaWcbaGaemOAaOgabeaakiabcMcaPiabgIGiolabdweafjabcIcaOiabdEeahjabcMcaPaqaaiabicdaWaqaaiabb+gaVjabbsha0jabbIgaOjabbwgaLjabbkhaYjabbEha3jabbMgaPjabbohaZjabbwgaLbqaaaaaaiaawUhaaaaa@6BFF@

The eigenvalues of the *Laplacian matrix *of a graph is the graph's *spectrum*. The eigenvalues are related to the density distribution of the edge set. The second smallest eigenvalue, denoted by *λ*_2 _(often called the Fiedler eigenvalue) is the best measure of the graph's connectivity among all of the eigenvalues. Large values for *λ*_2 _correspond to vertices of high degree that are in close proximity whereas small values for *λ*_2 _correspond to a more equally dispersed edge set.

### Domination based parameters

We calculated a number of graphical invariants for each tree and tabulated the results. As in the RAG database, the trees were cataloged by their Fiedler eigenvalues. In so doing, we noticed that the domination parameters behaved in two distinct ways with respect to the Fiedler eigenvalue. The domination, total domination, and global alliance numbers tended to decrease as the eigenvalues increased. The locating-domination and differentiating domination numbers tended to increase as the eigenvalues increased. Thus, we grouped the invariants into two classes and summed the values in each class. To normalize the results, the sums were divided by the total number of vertices in the tree, defining the two parameters *P*_1 _and *P*_2 _In the case where the invariants behaved oppositional to the eigenvalues, *P*_2 _was modified in the following way. Instead of dividing by the total number of vertices in the tree, the Fiedler eigenvalue was multiplied by the number of vertices and included the product in the sum. We denote this parameter by *P*_2*_. The three formulas for *P*_1_, *P*_1_, *P*_2 _and *P*_2* _are given below and are used to complete Table 1.

P1=γ+γt+γanP2=γL+γDnP2*=γL+γD+nλ2
 MathType@MTEF@5@5@+=feaafiart1ev1aaatCvAUfKttLearuWrP9MDH5MBPbIqV92AaeXatLxBI9gBaebbnrfifHhDYfgasaacH8akY=wiFfYdH8Gipec8Eeeu0xXdbba9frFj0=OqFfea0dXdd9vqai=hGuQ8kuc9pgc9s8qqaq=dirpe0xb9q8qiLsFr0=vr0=vr0dc8meaabaqaciaacaGaaeqabaqabeGadaaakqaabeqaaiabdcfaqnaaBaaaleaacqaIXaqmaeqaaOGaeyypa0ZaaSaaaeaaiiGacqWFZoWzcqGHRaWkcqWFZoWzdaWgaaWcbaGaemiDaqhabeaakiabgUcaRiab=n7aNnaaBaaaleaacqWGHbqyaeqaaaGcbaGaemOBa4gaaaqaaiabdcfaqnaaBaaaleaacqaIYaGmaeqaaOGaeyypa0ZaaSaaaeaacqWFZoWzdaWgaaWcbaacbiGae4htaWeabeaakiabgUcaRiab=n7aNnaaBaaaleaacqWGebaraeqaaaGcbaGaemOBa4gaaaqaaiabdcfaqnaaDaaaleaacqaIYaGmaeaacqGGQaGkaaGccqGH9aqpcqWFZoWzdaWgaaWcbaGaemitaWeabeaakiabgUcaRiab=n7aNnaaBaaaleaacqWGebaraeqaaOGaey4kaSIaemOBa4Mae83UdW2aaSbaaSqaaiabikdaYaqabaaaaaa@56FE@

As seen above, *P*_1 _is the sum of the graphical invariants that tended to decrease as the Fiedler eigenvalues increased. The other case is given by *P*_2 _and P2*
 MathType@MTEF@5@5@+=feaafiart1ev1aaatCvAUfKttLearuWrP9MDH5MBPbIqV92AaeXatLxBI9gBaebbnrfifHhDYfgasaacH8akY=wiFfYdH8Gipec8Eeeu0xXdbba9frFj0=OqFfea0dXdd9vqai=hGuQ8kuc9pgc9s8qqaq=dirpe0xb9q8qiLsFr0=vr0=vr0dc8meaabaqaciaacaGaaeqabaqabeGadaaakeaacqWGqbaudaqhaaWcbaGaeGOmaidabaGaeiOkaOcaaaaa@2FD0@.

### Statistical methods

Logistic models were used to predict the probability that a tree is a native RNA structure based on its domination numbers. Two different logistic models were estimated using SAS, one based on *P*_1 _and *P*_2_(definitions based on domination only) and another one based on *P*_1 _and P2*
 MathType@MTEF@5@5@+=feaafiart1ev1aaatCvAUfKttLearuWrP9MDH5MBPbIqV92AaeXatLxBI9gBaebbnrfifHhDYfgasaacH8akY=wiFfYdH8Gipec8Eeeu0xXdbba9frFj0=OqFfea0dXdd9vqai=hGuQ8kuc9pgc9s8qqaq=dirpe0xb9q8qiLsFr0=vr0=vr0dc8meaabaqaciaacaGaaeqabaqabeGadaaakeaacqWGqbaudaqhaaWcbaGaeGOmaidabaGaeiOkaOcaaaaa@2FD0@ (that considers domination and the second smallest eigenvalue). Due to the abrupt change from *native *to *not RNA-like *for small changes in *P*_1 _and *P*_2 _or *P*_1 _and P2*
 MathType@MTEF@5@5@+=feaafiart1ev1aaatCvAUfKttLearuWrP9MDH5MBPbIqV92AaeXatLxBI9gBaebbnrfifHhDYfgasaacH8akY=wiFfYdH8Gipec8Eeeu0xXdbba9frFj0=OqFfea0dXdd9vqai=hGuQ8kuc9pgc9s8qqaq=dirpe0xb9q8qiLsFr0=vr0=vr0dc8meaabaqaciaacaGaaeqabaqabeGadaaakeaacqWGqbaudaqhaaWcbaGaeGOmaidabaGaeiOkaOcaaaaa@2FD0@, the maximum likelihood estimation process does not converge; however the predicted categories obtained with those models were correct in 100% of the cases considering the 11 trees that are known to exist as RNA structures. The estimated values for the parameters correspond to the last iteration. Logistic models are usually evaluated by the percent of concordant pairs and the percent of correctly predicted values; for the two models the percent of concordant pairs and the percent of correct predictions (for those known to be native or predicted by RAG as 'not RNA-like') is 100%. The two models are:

Model 1 : ln[p^
 MathType@MTEF@5@5@+=feaafiart1ev1aaatCvAUfKttLearuWrP9MDH5MBPbIqV92AaeXatLxBI9gBaebbnrfifHhDYfgasaacH8akY=wiFfYdH8Gipec8Eeeu0xXdbba9frFj0=OqFfea0dXdd9vqai=hGuQ8kuc9pgc9s8qqaq=dirpe0xb9q8qiLsFr0=vr0=vr0dc8meaabaqaciaacaGaaeqabaqabeGadaaakeaacuWGWbaCgaqcaaaa@2E25@/(1 - p^
 MathType@MTEF@5@5@+=feaafiart1ev1aaatCvAUfKttLearuWrP9MDH5MBPbIqV92AaeXatLxBI9gBaebbnrfifHhDYfgasaacH8akY=wiFfYdH8Gipec8Eeeu0xXdbba9frFj0=OqFfea0dXdd9vqai=hGuQ8kuc9pgc9s8qqaq=dirpe0xb9q8qiLsFr0=vr0=vr0dc8meaabaqaciaacaGaaeqabaqabeGadaaakeaacuWGWbaCgaqcaaaa@2E25@)] = -146.1 + 104.3*P*_1 _+ 16.6148*P*_2_

Model 2 : ln[p^
 MathType@MTEF@5@5@+=feaafiart1ev1aaatCvAUfKttLearuWrP9MDH5MBPbIqV92AaeXatLxBI9gBaebbnrfifHhDYfgasaacH8akY=wiFfYdH8Gipec8Eeeu0xXdbba9frFj0=OqFfea0dXdd9vqai=hGuQ8kuc9pgc9s8qqaq=dirpe0xb9q8qiLsFr0=vr0=vr0dc8meaabaqaciaacaGaaeqabaqabeGadaaakeaacuWGWbaCgaqcaaaa@2E25@/(1 - p^
 MathType@MTEF@5@5@+=feaafiart1ev1aaatCvAUfKttLearuWrP9MDH5MBPbIqV92AaeXatLxBI9gBaebbnrfifHhDYfgasaacH8akY=wiFfYdH8Gipec8Eeeu0xXdbba9frFj0=OqFfea0dXdd9vqai=hGuQ8kuc9pgc9s8qqaq=dirpe0xb9q8qiLsFr0=vr0=vr0dc8meaabaqaciaacaGaaeqabaqabeGadaaakeaacuWGWbaCgaqcaaaa@2E25@)] = -145.3 + 106.1*P*_1 _+ 1.4908P2*
 MathType@MTEF@5@5@+=feaafiart1ev1aaatCvAUfKttLearuWrP9MDH5MBPbIqV92AaeXatLxBI9gBaebbnrfifHhDYfgasaacH8akY=wiFfYdH8Gipec8Eeeu0xXdbba9frFj0=OqFfea0dXdd9vqai=hGuQ8kuc9pgc9s8qqaq=dirpe0xb9q8qiLsFr0=vr0=vr0dc8meaabaqaciaacaGaaeqabaqabeGadaaakeaacqWGqbaudaqhaaWcbaGaeGOmaidabaGaeiOkaOcaaaaa@2FD0@

where p^
 MathType@MTEF@5@5@+=feaafiart1ev1aaatCvAUfKttLearuWrP9MDH5MBPbIqV92AaeXatLxBI9gBaebbnrfifHhDYfgasaacH8akY=wiFfYdH8Gipec8Eeeu0xXdbba9frFj0=OqFfea0dXdd9vqai=hGuQ8kuc9pgc9s8qqaq=dirpe0xb9q8qiLsFr0=vr0=vr0dc8meaabaqaciaacaGaaeqabaqabeGadaaakeaacuWGWbaCgaqcaaaa@2E25@ is the estimated probability of being native given the values of *P*_1 _and *P*_2 _or *P*_1 _and P2*
 MathType@MTEF@5@5@+=feaafiart1ev1aaatCvAUfKttLearuWrP9MDH5MBPbIqV92AaeXatLxBI9gBaebbnrfifHhDYfgasaacH8akY=wiFfYdH8Gipec8Eeeu0xXdbba9frFj0=OqFfea0dXdd9vqai=hGuQ8kuc9pgc9s8qqaq=dirpe0xb9q8qiLsFr0=vr0=vr0dc8meaabaqaciaacaGaaeqabaqabeGadaaakeaacqWGqbaudaqhaaWcbaGaeGOmaidabaGaeiOkaOcaaaaa@2FD0@. When p^
 MathType@MTEF@5@5@+=feaafiart1ev1aaatCvAUfKttLearuWrP9MDH5MBPbIqV92AaeXatLxBI9gBaebbnrfifHhDYfgasaacH8akY=wiFfYdH8Gipec8Eeeu0xXdbba9frFj0=OqFfea0dXdd9vqai=hGuQ8kuc9pgc9s8qqaq=dirpe0xb9q8qiLsFr0=vr0=vr0dc8meaabaqaciaacaGaaeqabaqabeGadaaakeaacuWGWbaCgaqcaaaa@2E25@ > 0.5 the tree is predicted to be native. The values of p^
 MathType@MTEF@5@5@+=feaafiart1ev1aaatCvAUfKttLearuWrP9MDH5MBPbIqV92AaeXatLxBI9gBaebbnrfifHhDYfgasaacH8akY=wiFfYdH8Gipec8Eeeu0xXdbba9frFj0=OqFfea0dXdd9vqai=hGuQ8kuc9pgc9s8qqaq=dirpe0xb9q8qiLsFr0=vr0=vr0dc8meaabaqaciaacaGaaeqabaqabeGadaaakeaacuWGWbaCgaqcaaaa@2E25@ obtained with each one of the two models are very similar and therefore the predictions as *native *or *not RNA-like *for both models are the same for each the trees. All of the 23 trees whose status is either 'native' or 'RAG predicted not RNA-like' were likewise predicted by the two domination models. From the 4 RAG candidates with 7 vertices (RAG RNA-like, but not yet found as a native structure), 3 are predicted by the domination model as RNA-like and one, (7.4), as non-RNA like. From the 8 candidates with 8 vertices, 7 are predicted to be native and only one, (8.13), is predicted as not RNA-like. Table 1 displays the values of *P*_1_, *P*_2 _and P2*
 MathType@MTEF@5@5@+=feaafiart1ev1aaatCvAUfKttLearuWrP9MDH5MBPbIqV92AaeXatLxBI9gBaebbnrfifHhDYfgasaacH8akY=wiFfYdH8Gipec8Eeeu0xXdbba9frFj0=OqFfea0dXdd9vqai=hGuQ8kuc9pgc9s8qqaq=dirpe0xb9q8qiLsFr0=vr0=vr0dc8meaabaqaciaacaGaaeqabaqabeGadaaakeaacqWGqbaudaqhaaWcbaGaeGOmaidabaGaeiOkaOcaaaaa@2FD0@ for all trees with 7 and 8 vertices. The estimated probability of being native p^
 MathType@MTEF@5@5@+=feaafiart1ev1aaatCvAUfKttLearuWrP9MDH5MBPbIqV92AaeXatLxBI9gBaebbnrfifHhDYfgasaacH8akY=wiFfYdH8Gipec8Eeeu0xXdbba9frFj0=OqFfea0dXdd9vqai=hGuQ8kuc9pgc9s8qqaq=dirpe0xb9q8qiLsFr0=vr0=vr0dc8meaabaqaciaacaGaaeqabaqabeGadaaakeaacuWGWbaCgaqcaaaa@2E25@ obtained with each one of the models (ml and m2) and the RAG status are also displayed.

A third model, using *P*_1 _as sole predictor was also estimated. Again the estimation process does not converge because of the abrupt change and total separation of values; *P*_1 _< 1.2 for all natives and *P*_1 _> 1.2 for all not RNA like. The model with the estimates of the last iteration is ln[p^
 MathType@MTEF@5@5@+=feaafiart1ev1aaatCvAUfKttLearuWrP9MDH5MBPbIqV92AaeXatLxBI9gBaebbnrfifHhDYfgasaacH8akY=wiFfYdH8Gipec8Eeeu0xXdbba9frFj0=OqFfea0dXdd9vqai=hGuQ8kuc9pgc9s8qqaq=dirpe0xb9q8qiLsFr0=vr0=vr0dc8meaabaqaciaacaGaaeqabaqabeGadaaakeaacuWGWbaCgaqcaaaa@2E25@/(1 - p^
 MathType@MTEF@5@5@+=feaafiart1ev1aaatCvAUfKttLearuWrP9MDH5MBPbIqV92AaeXatLxBI9gBaebbnrfifHhDYfgasaacH8akY=wiFfYdH8Gipec8Eeeu0xXdbba9frFj0=OqFfea0dXdd9vqai=hGuQ8kuc9pgc9s8qqaq=dirpe0xb9q8qiLsFr0=vr0=vr0dc8meaabaqaciaacaGaaeqabaqabeGadaaakeaacuWGWbaCgaqcaaaa@2E25@)] = -109.3 + 91.407*P*_1 _The estimated probabilities *P*(*native*) are plotted for a range of values of *P*_1 _in Figure 7. The class (native or not RNA-like) predicted for the candidates using this model is the same given by the other two models.

## Authors' contributions

Teresa Haynes provided guidance on the selection of the graphical invariants and assisted in the calculation of their values. Debra Knisley conceived the project and is the primary author. She worked with Teresa Haynes on the graphical invariants and the determination of the resulting parameters. Edith Seier provided the statistical analysis and Yue Zoe provided his expertise in the biochemistry of RNA.

## Figures and Tables

**Table 1:** Status and prediction for trees with seven and eight vertices

*Vertices*	ID	*P*_1_	*P*_2_	P2* MathType@MTEF@5@5@+=feaafiart1ev1aaatCvAUfKttLearuWrP9MDH5MBPbIqV92AaeXatLxBI9gBaebbnrfifHhDYfgasaacH8akY=wiFfYdH8Gipec8Eeeu0xXdbba9frFj0=OqFfea0dXdd9vqai=hGuQ8kuc9pgc9s8qqaq=dirpe0xb9q8qiLsFr0=vr0=vr0dc8meaabaqaciaacaGaaeqabaqabeGadaaakeaacqWGqbaudaqhaaWcbaGaeGOmaidabaGaeiOkaOcaaaaa@2FD0@	P(Native) *model*1	*P*(*Native*) *model*2	RAG Status	Domination Predicted status
7	1	1.57143	1.00000	8.3867	1.00000	1.00000	*native*	*native*
7	2	1.28571	1.28571	10.5778	0.99898	0.99991	*native*	*native*
7	3	1.42857	1.00000	8.8221	1.00000	1.00000	*native*	*native*
7	4	1.14286	1.28571	10.8753	0.00040	0.00392	*candidate*	*not RNA like*
7	5	1.28571	1.28571	11.0685	0.99951	0.99991	*candidate*	*native*
7	6	1.28571	1.14286	10.2519	0.99834	0.99908	*native*	*native*
7	7	1.28571	1.14286	10.6740	0.99911	0.99908	*candidate*	*native*
7	8	1.57143	1.00000	9.6740	1.00000	1.00000	*candidate*	*native*
7	9	1.00000	1.42857	12.7881	0.00000	0.00000	*not RNA like*	*not RNA like*
7	10	1.00000	1.42857	13.2613	0.00000	0.00000	*not RNA like*	*not RNA like*
7	11	1.00000	1.71429	19.0000	0.00002	0.00000	*not RNA like*	*not RNA like*
8	1	1.37500	1.12500	10.2176	1.00000	1.00000	*candidate*	*native*
8	2	1.37500	1.12500	10.3336	1.00000	1.00000	*candidate*	*native*
8	3	1.37500	1.12500	10.4912	1.00000	1.00000	*native*	*native*
8	4	1.25000	1.25000	11.4912	0.98853	0.99359	*candidate*	*native*
8	5	1.50000	1.00000	9.5848	1.00000	1.00000	*native*	*native*
8	6	1.25000	1.25000	11.6184	0.99049	0.99359	*candidate*	*native*
8	7	1.37500	1.12500	10.7096	1.00000	1.00000	*native*	*native*
8	8	1.25000	1.12500	10.7944	0.96824	0.95104	*candidate*	*native*
8	9	1.12500	1.37500	12.9072	0.00124	0.00269	*not RNA like*	*not RNA like*
8	10	1.50000	1.12500	10.9472	1.00000	1.00000	*native*	*native*
8	11	1.50000	1.00000	10.0072	1.00000	1.00000	*native*	*native*
8	12	1.37500	1.12500	11.0304	1.00000	1.00000	*candidate*	*native*
8	13	1.12500	1.25000	12.1432	0.00040	0.00034	*candidate*	*not RNA like*
8	14	1.12500	1.37500	13.2192	0.00196	0.00269	*not RNA like*	*not RNA like*
8	15	1.25000	1.25000	12.3104	0.99659	0.99359	*native*	*native*
8	16	1.37500	1.12500	11.4520	1.00000	1.00000	*candidate*	*native*
8	17	1.12500	1.25000	12.5496	0.00073	0.00034	*not RNA like*	*not RNA like*
8	18	1.00000	1.50000	14.8336	0.00000	0.00000	*not RNA like*	*not RNA like*
8	19	0.87500	1.50000	14.9904	0.00000	0.00000	*not RNA like*	*not RNA like*
8	20	1.50000	1.00000	11.0560	1.00000	1.00000	*native*	*native*
8	21	1.12500	1.25000	13.0560	0.00154	0.00034	*not RNA like*	*not RNA like*
8	22	1.00000	1.50000	15.6200	0.00000	0.00000	*not RNA like*	*not RNA like*
8	23	0.87500	1.75000	22.0000	0.00000	0.00000	*not RNA like*	*not RNA like*
